# Migraine or Acute Angle Closure?

**Published:** 2020

**Authors:** Cristina Stan, Cristina Stan, Anca Maria Rednik

**Affiliations:** * “Iuliu Haţieganu” University of Medicine and Pharmacy Cluj-Napoca, Cluj, Romania; ** Department of Ophthalmology, County Emergency Hospital Cluj-Napoca, Cluj, Romania

**Keywords:** aura, migraine, acute angle closure

## Abstract

This paper aimed to present a relatively frequent misinterpretation of a migraine with visual aura. Sometimes, patients with aura and migraine are referred to the ophthalmologic unit with the diagnosis of acute angle closure attack. Thus, we discussed the way an ophthalmologist could make a difference between these two entities.

## Introduction

Two types of migraine that go along with visual disturbances have been described in literature: the ocular migraine and the migraine with aura.

The retinal migraine (otherwise known as ophthalmic migraine, anterior visual pathway migraine, or ocular migraine) occurs with unilateral visual loss accompanied or followed by headache. The episode of visual impairment is usually of shorter duration than the one in migraine-associated aura. The pathogenic mechanism of the ocular migraine appears to be retinal vasospasm [**1**]. Patients suffering from ocular migraine accuse symptoms that include complete visual loss (50%) or incomplete visual loss (12%), blurring (19%), dimming (7%), scotomas, central visual loss or negative scotoma and altitudinal field defects (13%) [**1**].

The visual disturbance in the migraine with aura consists of a bilateral positive scotoma that more often involves the peripheral visual field (implying a cortical origin) [**1**]. Characteristically, the scotoma from aura is mobile and shiny. Patients often wrongly perceive a homonymous visual field defect as a unilateral issue. The visual symptoms gradually disappear with complete resolution and the migraine usually starts simultaneously with the resolution of the scotoma. In few cases, the aura is not followed by headache, this usually happening in elderly patients [**2**].

## Case report

We examined several cases with the ”refferal diagnosis” of acute angle closure attack.

1. 16-year-old male with positive brilliant scotomas that occured three times in one month with the duration of 5-6 minutes, followed by unilateral migraine; no trigger was found; no other diseases.

2. 66-year-old female, one episode of blurred vision with ”moving lights in one eye” followed by ipsilateral migraine; the episode repeated after 6 months; no visual simptoms between the episodes and no known trigger event.

3. 44-year-old female, one episode of blurred vision, with ”moving lights in one eye” followed by ipsilateral migraine.

4. 23-year-old female, one episode of ”zig-zag brilliant thick line” followed by ”patches of visual loss” and unilateral ”horrible migraine”. 

## Results

The ophthalmological exam was normal in all cases. 

Gonioscopy showed a large, open angle.

Our diagnosis was ”migraine with aura” and we reffered the patients to the neurologist.

## Discussion 

Why is there the confusion between the ocular migraine and migraine with aura, and on the other side, the acute angle closure attack?

Both include symptoms such as visual disturbances and unilateral headache. But the visual disturbances manifest differently. In the migraine with aura, there are brilliant and moving scotomas followed by localized scotomas (for example, the patient can see his palm, but only some parts of his fingers). Binocular scintillations that expand gradually, scotomas in the form of ”scintillating scotoma” or ”fortification scotoma” (“zig-zag lines”) that usually last more than five 5 minutes and up to 20 minutes but always less than 60 minutes, are specific for a migrainous visual aura, especially if a headache precedes the aura. The characteristic visual aura of a migraine appears in a hemifield rather than the whole field of a single eye [**3**]. Imaging studies and laboratory tests should exclude ocular pathologies, neurologic disease (seizures and central nervous system tumor), thrombosis or carotid or cardiac disease, and connective tissue disease [**4**].

The International Headache Society (IHS) describes the ”retinal migraine” as monocular attacks of visual impairment that are fully reversible combined with migraine headaches in the context of a normal neuroophthalmic assesment between the episodes [**3**]. Negative monocular visual event with a duration of maximum 1 hour and migrain headache on the same side as the visual loss is characteristic [**5**]. If unilateral visual loss is proved, one must exclude all causes of permanent or transient monocular visual loss; retinal/ ocular migraine is a diagnosis of exclusion [**5**]. 

In acute angle closure attack, the vision is totally blurred with the perception of rainbows that do not move around the light souces. The most important ophthalmologic exam is gonioscopy: if the anterior chamber angle is wide open (grade 3 or 4 Schaffer), the diagnosis of angle closure attack is infirmed. If the anterior chamber angle is narrow, one should listen carefully to the patients’ medical history because the description of the scotoma will aid the diagnosis. However, the two diseases could co-exist at the same time, in the same patient.

The pathophysiology of migraines remains controversial. Arterial vasospasm has been shown to be the physiopathological mechanism of the visual loss in ocular migraine, leaving little room for debate [**6**]. On the other hand, the aura of migraine is now thought to come from the cortical “spreading depression” (SD) (Leao’s theory) leading to the release of a wave of activity that arises from the visual cortex and advances between 2 and 3 mm/ min. Once axons are excited, neurogenic and vascular inflammation ensue with soft tissue swelling as seen in the cerebral cortex, retina, optic disc, and peripherally in the temple and conjunctiva. Alternatively, inflammation that interests the ophthalmic and retinal vessels may lead to vasospasm, obstruction of the vessel, and/ or ocular sympathetic dysfunction that could explain the visual symptoms [**1**]. Functional imaging and magneto encephalographic studies identified the cortical SD as the biological basis in the occipital aura prior to the headache in migraines [**3**]. A chracteristic of the visual aura of migraine is that it occurs in a hemifield, as a result of its origin in the primary visual cortex in the occipital lobe [**3**].

## Conclusion

1. Not every headache with blurred vision is acute glaucoma.

2. Differential diagnostic needs detailed history + gonioscopy.

3. The doctor should carefully listen to what the patient describes in order to differentiate ocular migraine from migraine with aura.
